# Multiple superovulations alter histone modifications in mouse early embryos

**DOI:** 10.1530/REP-18-0495

**Published:** 2019-03-18

**Authors:** Shou-Bin Tang, Lei-Lei Yang, Ting-Ting Zhang, Qian Wang, Shen Yin, Shi-Ming Luo, Wei Shen, Zhao-Jia Ge, Qing-Yuan Sun

**Affiliations:** 1 College of Animal Science and Technology, Institute of Reproductive Sciences, Qingdao Agricultural University, Qingdao, People’s Republic of China; 2 Reproductive Medicine Center of People’s Hospital of Zhengzhou University, Zhengzhou, Henan Province, People’s Republic of China; 3 College of Life Sciences, Institute of Reproductive Sciences, Qingdao Agricultural University, Qingdao, People’s Republic of China; 4 State Key Laboratory of Stem Cell and Reproductive Biology, Institute of Zoology, Chinese Academy of Sciences, Beijing, People’s Republic of China

## Abstract

It is demonstrated that repeated superovulation has deleterious effects on mouse ovaries and cumulus cells. However, little is known about the effects of repeated superovulation on early embryos. Epigenetic reprogramming is an important event in early embryonic development and could be easily disrupted by the environment. Thus, we speculated that multiple superovulations may have adverse effects on histone modifications in the early embryos. Female CD1 mice were randomly divided into four groups: (a) spontaneous estrus cycle (R0); (b) with once superovulation (R1); (c) with three times superovulation at a 7-day interval (R3) and (d) with five times superovulation at a 7-day interval (R5). We found that repeated superovulation remarkably decreased the fertilization rate. With the increase of superovulation times, the rate of early embryo development was decreased. The expression of *Oct4*, *Sox2* and *Nanog* was also affected by superovulation in blastocysts. The immunofluorescence results showed that the acetylation level of histone 4 at lysine 12 (H4K12ac) was significantly reduced by repeated superovulation in mouse early embryos (*P* < 0.01). Acetylation level of histone 4 at lysine 16 (H4K16ac) was also significantly reduced in pronuclei and blastocyst along with the increase of superovulation times (*P* < 0.01). H3K9me2 and H3K27me3 were significantly increased in four-cell embryos and blastocysts. We further found that repeated superovulation treatment increased the mRNA level of histone deacetylases *Hdac1*, *Hdac2* and histone methyltransferase G9a, but decreased the expression level of histone demethylase-encoding genes *Kdm6a* and *Kdm6b* in early embryos. In a word, multiple superovulations alter histone modifications in early embryos.

## Introduction

With rapid socio-economic development, more and more young people have reproductive health problems that are induced by many factors such as environmental pollution, unhealthy lifestyle and increasing incidence of chronic diseases. Approximately, 10–15% couples worldwide at childbearing age suffer from infertility and sterility. The use of ARTs (assisted reproductive techniques) for the treatment of human infertility/subfertility is rapidly increasing, although the successful incidence is not as high as expected. Until now, ART is the best treatment for infertility. Approximately, 5–10% of newborn babies each year are produced by ART in some developed countries (Sunderam *et al.* 2015, European IVF-Monitoring Consortium *et al.* 2016). Thus, the health of ART children has become a major concern. Previous studies reported that a high frequency of chromosomal abnormalities (Van Steirteghem *et al.* 2002), rare congenital malformations (Hansen *et al.* 2002, Bonduelle *et al.* 2005) and alterations of cognitive and motor development (Stromberg *et al.* 2002, Kallen *et al.* 2005) in children may be associated with ART, but how that happens is not clear.

Epigenetic status changes saliently during preimplantation embryo development and gametogenesis in which epigenetic modifications are sensitive to environmental changes (Erhardt *et al.* 2003, van der Heijden *et al.* 2005, Wang & Dey 2006, Zhang *et al.* 2009). In recent years, a number of studies have shown that ART manipulations such as superovulation, vitrification and *in vitro culture* can induce changes of epigenetic modifications in embryos and fetus (El Hajj & Haaf 2013, Ventura-Junca *et al.* 2015). For example, superovulation and vitrification alter H4K12ac (histone 4 lysine 12 acetylation) and H3K9ac (histone 3 lysine 9 acetylation) in ICM (inner cell mass) and TE (trophectoderm) (Bakhtari *et al.* 2014), and superovulation affects DNA methylation pattern of* line-1* in blastocyst (Liang *et al.* 2013). In the clinic, many women would experience more than one exogenous hormone-stimulated cycle before getting a baby. Animal studies have demonstrated that multi-superovulation alters ovarian structure and function in the rhesus monkey, as well as mitochondrial distribution and function in mouse ovaries and cumulus cells (Chao *et al.* 2005, Dong *et al.* 2014, Kalthur *et al.* 2016, Xie *et al.* 2016). Although the influence of superovulation on DNA methylation is dose dependent (Market-Velker *et al.* 2010), the effect of repeated superovulation at low dose on epigenetics in embryos is still not well known. So we hypothesized that repeated superovulation may have adverse effects on epigenetic modifications in embryos.

Histone modification is one of the most important epigenetic modifications, which plays a critical role in early embryonic development (Adenot *et al.* 1997, Li 2002, Morgan *et al.* 2005, Santos *et al.* 2005, Bogliotti & Ross 2012, Aoshima *et al.* 2015). It is demonstrated that H3K4me3 (tri-methylation at histone 3 lysine 4) and H3K27me3 (tri-methylation at histone 3 lysine 27) are reprogrammed in early embryos, which is very important for embryo development (Liu *et al.* 2016, Zhang *et al.* 2016). H3K9me2 (di-methylation at histone 3 lysine 9) is an important marker of heterochromatin which can repress the genes expression (Sridharan *et al.* 2013). H3K9me2 regulates DNA methylation by recruiting PGC7 to chromatin in the early embryos (Nakamura *et al.* 2012). There is a dynamic change of H3K9me2 in early embryonic development. H4K12ac is associated with cell division (Shang *et al.* 2016) and is important for chromatin decondensation in zygotes and early embryonic development (van der Heijden *et al.* 2006, Paradowska *et al.* 2012). H4K16ac is involved in chromatin structure remodeling (Grigoryan *et al.* 2018) and early embryonic development (van der Heijden *et al.* 2006). So we tested the effects of multi-superovulation on histone modifications such as H4K12ac, H4K16ac, H3K27me3 and H3K9me2 in the early embryos. We found that repeated superovulations altered histone modifications in the early embryos. To elucidate how these happen, we further examined the mRNA expression of histone deacetylases (*Hdac1*, *Hdac2*), acetylases (*Gnc5*, *Hat1*), methyltransferase (*G9a*) and histone demethylases (*Kdm6a*, *Kdm6b*) in the early embryos.

## Materials and methods

### Ethics statement

Animal care and use were conducted in accordance with the guideline of Qingdao Agricultural University, China. Mice were housed in a temperature-controlled room with proper darkness–light cycles and fed a regular diet. All experiments and the study protocol were approved by the Ethics Committee of Qingdao Agricultural University.

### Superovulation

**Figure 1:**
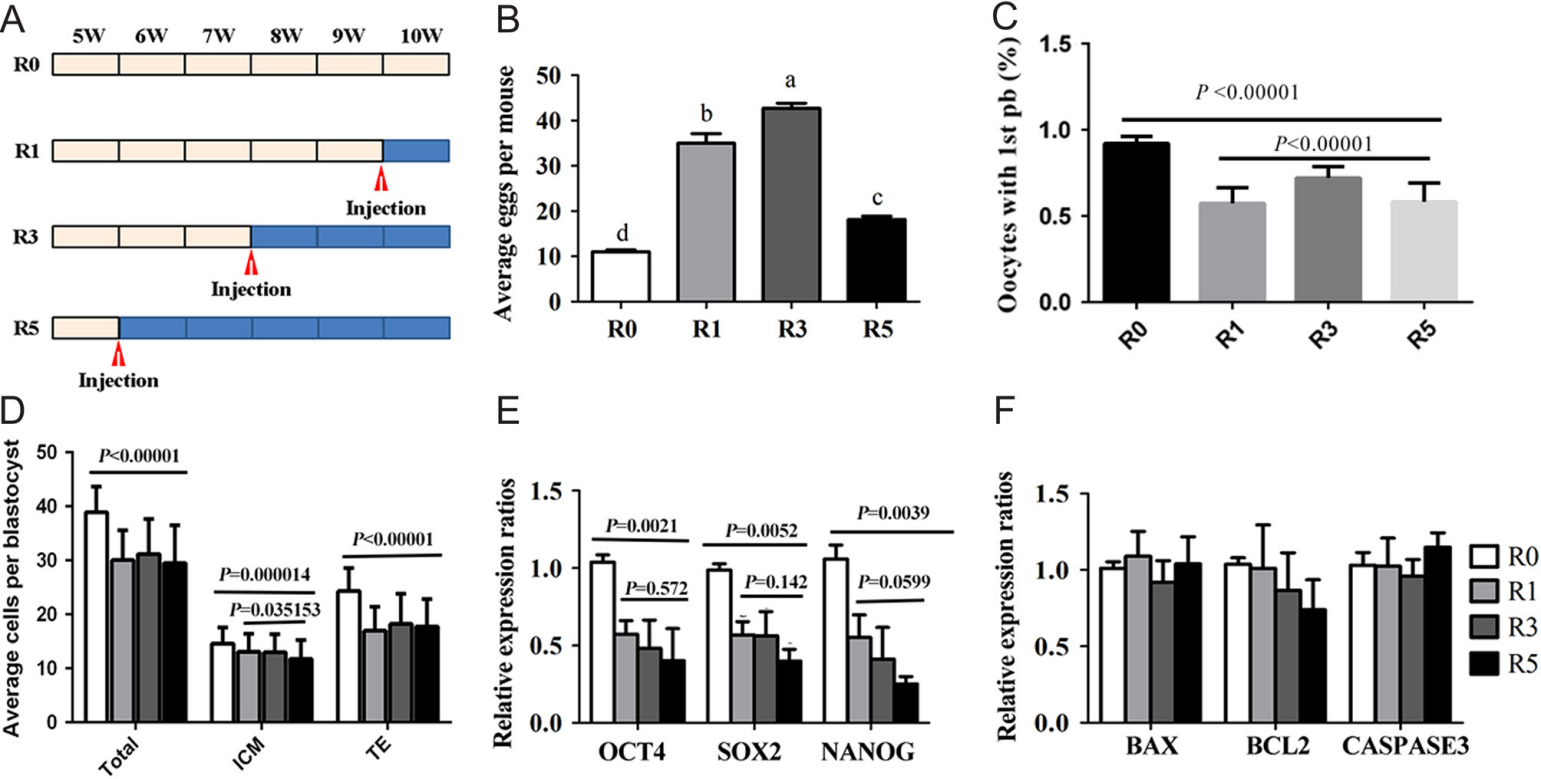
Effects of multiple superovulations on embryonic development. (A) Schedule of superovulation; (B) average number of eggs retrieved from each mouse after mating with males; (C) the incidence of oocytes with the 1st PB; (D) the cell number of blastocysts. The number of blastocysts tested was 67, 73, 64 and 74 in R0, R1, R3 and R5 groups, respectively. (E and F) Effects of multiple superovulations on embryonic quality were also examined via checking the expression of the genes, such as pluripotency-related genes *Oct4*, *Sox2*, *Nanog* and apoptosis-related genes *Bcl2*, *Bax* and *Casp3*.

Female CD1 mice (5 weeks of age) were purchased from the Center of Experimental Animals of Qingdao and fed in a temperature- and humidity-controlled room at a light cycle of 12 h light and 12 h darkness. Diet and water were supplied *ad libitum*. Female CD1 mice were randomly divided into four groups (Fig. 1A): (a) natural estrus cycle (R0); (b) intraperitoneal injection with 8 IU PMSG (Ningbo Hormone Product Co. Ltd., China) and 8 IU hCG (Ningbo Hormone Product Co. Ltd., China) for once (R1); (c) intraperitoneal injection with 8 IU PMSG and 8 IU hCG for three times at a 7-day interval (R3); (d) intraperitoneal injection with 8 IU PMSG and 8 IU hCG for five times at a 7-day interval (R5).

### Zygote collection and embryo culture

Females were mated with fertile males to produce zygotes at the last time of hCG injection. The same males were used in each treatment, and all the males were purchased from the Center of Experimental Animals of Qingdao. If the virginal plug was observed in the next morning, females were killed by cervical dislocation 20 h after hCG administration. Fertilized eggs surrounded with cumulus cells were collected from oviduct ampulla. After that, cumulus cells were removed using 1 mg/mL hyaluronidase in the M2 medium. To collect fertilized eggs of R0, pre-estrus mice were selected and mated with males. Then, fertilized eggs from the above four groups were cultured in KSOM + AA culture medium under liquid paraffin oil at 37°C with 5% CO_2_. We obtained PN4 (8 h) fertilized eggs, two-cell-stage embryos (24 h), four-cell-stage embryos (48 h) and blastocysts (84 h and 108 h).

### Antibodies and immunofluorescence

Antibodies for detecting H4K12ac (catalog No. 39927) and H4K16ac (catalog No. 39727) were purchased from Santa Cruz Biotechnology. The antibody of H3K9me2 was purchased from Bioworld Technology (catalog No. BS7234) and H3K27me3 was purchased from Abcam (catalog No. ab6002). Antibodies of FITC (catalog No. FITC1) and Cy3 (catalog No. L0419) were purchased from Sigma. Immunofluorescence was performed according to previous protocols (Ma *et al.* 2014). Briefly, embryos were fixed in 4% paraformaldehyde for 40 min and then treated with 0.5% Triton X-100 for 20 min after three washes using washing buffer (PBS with 0.1% Tween 20 and 0.01% Triton-100). After blocking using 1% BSA for 1 h, embryos were incubated with the primary antibody (1:50) overnight at 4°C. After washing five times using washing buffer, embryos were then incubated with the secondary antibody (1:200) for 2 h at room temperature. Then, embryos were incubated with Hoechst 33342 (propidium iodide/4,6-diamino-2-phenylindole) for 20 min to counterstain DNA after washing three times using washing buffer. Finally, the embryos were mounted on glass slides with DABCO and examined using a laser scanning confocal microscope (Leica TCS SP5).

### Quantitative fluorescence intensity

Stained embryos were scanned using the Leica TCS SP5 confocal microscope. For each antibody detection, the same excitation wavelength was used, including FITC excitation wavelength of 488 nm, Cy3 excitation wavelength of 561 nm. We unified the background value in the process of laser scanning. To quantify fluorescence intensity, we put all the Z-stacks together. The fluorescence intensity was analyzed and treated using ImageJ.

### Calculation of blastocyst cells

Cell number of blastocysts was counted as previously described (Van der Elst *et al.* 1998). Briefly, blastocysts were incubated in rabbit anti-mouse serum for 30 min, and then incubated in solution supplemented with 1:5 guinea-pig serum and PI for 5 min. After that, blastocysts were fixed using 4% paraformaldehyde for 40 min, and then treated with 0.5% Triton X-100 for 20 min. Then, blastocysts were incubated with Hoechst 33342 for 20 min after washing three times using washing buffer. Finally, the blastocysts were mounted on glass slides with DABCO. The TE and ICM cells were stained in red and blue, respectively. Blastocysts were scanned using a laser scanning confocal microscope (Leica TCS SP5). The cell numbers were counted using ImageJ.

### RNA extraction and quantitative real-time PCR (qRT-PCR)

Total RNA was extracted using EZ-10 Spin Column Total RNA Isolation Kit (Sangon Biotech, Shanghai, China) according to the manufacturer’s instruction. A total of 50–150 embryos were collected for the RNA extraction of each sample, depending on their developmental stages. cDNA was synthesized using the HiScript IIQ RT SuperMix (Vazyme, Nanjing, China) according to the manufacturer’s instructions. The synthesized cDNA was used as the template for qRT-PCR or stored at −80°C until used. Primers were shown in Table 1. qRT-PCR was carried out using the Applied Biosystems 7500 Sequence Detection System. Amplification was performed in the 20 μL volume containing 10 μL of SYBR Green Master Mix (Vazyme), 0.4 μL of primers (10 mM), 0.4 μL of ROX Reference Dye 2, 2 μL of cDNA and 7.2 μL of RNase free H_2_O. PCR amplification conditions were as follows: the reaction was initiated at 95°C for 10 min, followed by 40 cycles of denaturing at 95°C for 15 s, annealing at 60°C for 30 s and extension at 72°C for 20 s. Values were normalized against the expression level of GAPDH (reference). Relative expression values were calculated with the 2^−ΔΔCt^ method. Values of gene expression were means of three replicates.

**Table 1:** The sequence of primers used in RT-PCR.

Gene	Primer (5′–3′)		Size (bp)
	Forward	Reverse	
*Hdac1*	TTCCAACATGACCAACCA	AGCATCCTCAAGTTCTCAA	78
*Hdac2*	CCAGAACACTCCAGAATA	CATCTCCACTGTCTTCAT	131
*G9a*	TCATCTGCGAGTATGTAG	CGAAGAGGTAAGAATCATC	75
*Kdm6a (UTX)*	CAGTATAAGTTAGCAGTGGAA	GCGTTCTCAGAAGACAAT	157
*Kdm6b (Jmjd3)*	GACGAGCCTGCCTACTAC	TGCCATTCTCACTTGTAACG	76
*Hat1*	AAGTGTAACACCAACACAGCA	CGAAAGCAGTTTCATCATCCCC	127
*Gcn5*	AAGGCCAATGAAACCTGCAAG	CTCACAGCTACGGCACAACTC	117
*Oct4*	GGCTTCAGACTTCGCCTCC	AACCTGAGGTCCACAGTATGC	211
*Sox2*	GCGGAGTGGAAACTTTTGTCC	CGGGAAGCGTGTACTTATCCTT	157
*Nanog*	TCTTCCTGGTCCCCACAGTTT	GCAAGAATAGTTCTCGGGATGAA	100
*Bax*	ATGCGTCCACCAAGAAGCTGAG	CCCCAGTTGAAGTTGCCATCAG	166
*Bcl2*	ATGATAACCGGGAGATCGTG	GACGGTAGCGACGAGAGAAG	294
*Casp 3*	GACTGGGATGAACCACGACCC	TCTGACTGGAAAGCCGAAAC	205
*Gapdh*	GACAAAATGGTGAAGGTCGGT	GAGGTCAATGAAGGGGTCG	120

### Statistical analysis

The expression of genes, cell numbers and fluorescence intensity were represented as mean ± s.d. Differences were evaluated by one-way analysis of variance. The differences of data that were presented as a percentage were calculated with one-way ANOVA test. If the *P* value was <0.05, the difference between groups was considered significant.

## Results

### Repeated superovulation reduced the potency of early embryonic development

Zygotes were collected from oviducts 20 h after hCG injection and cultured *in vitro* to monitor the developmental process of early embryos. The average number of eggs per mouse (including fertilized and unfertilized) was higher in superovulated groups compared to that of R0 group, but it was decreased with the increase of superovulation times (*P* < 0.01, Fig. 1B). However, it appeared that the decrease in the percentage of oocytes with the first polar body (1st PB) was a consequence of repeated superovulation (*P* < 0.00001). Superovulation times had adverse effects on the percentage of oocytes with the 1st PB (Fig. 1C, *P* < 0.00001). The rate of pronucleus was higher in R0 group than that in R1, R3 and R5 groups (*P* < 0.01, Table 2). With the increase of superovulation times, there was a significant decrease in the rate of pronucleus formation in R1, R3 and R5 groups (*P* < 0.05, Table 2).

**Table 2:** Early embryonic development *in vitro*. Data are presented as percent ± s.d.

	*n*	8 h	24 h			48 h			84 h	108 h
		Pronucleus	Death	Block	2-Cell	Death	Block	4-Cell	Blastocyst	
R0	1956	91.33 ± 4.14^a^	1.79 ± 1.51^c^	1.73 ± 1.34^c^	97.95 ± 2.52^a^	8.83 ± 6.64^b^	3.18 ± 2.60^b^	94.55 ± 3.32^a^	26.57 ± 6.44^a^	82.92 ± 8.19^a^
R1	4195	50.40 ± 5.19^b^	4.51 ± 4.89^c^	8.16 ± 3.87^a^	91.85 ± 2.99^b^	28.55 ± 6.39^a^	6.78 ± 3.85^b^	80.14 ± 5.37^b^	12.34 ± 3.49^b^	51.98 ± 8.16^b^
R3	4973	66.44 ± 6.15^c^	12.03 ± 5.70^b^	4.11 ± 2.77^b^	91.73 ± 5.17^b^	27.53 ± 7.93^a^	14.53 ± 5.66^a^	77.03 ± 5.13^b^	5.53 ± 2.03^c^	44.76 ± 10.46^c^
R5	2025	42.73 ± 9.45^d^	21.04 ± 6.74^a^	8.01 ± 4.35^a^	85.39 ± 7.56^c^	11.85 ± 9.71^b^	11.85 ± 9.71^ab^	59.06 ± 9.31^c^	8.68 ± 4.94^bc^	37.72 ± 10.69^d^

Different letters mean *P* value <0.05 between groups. Pronucleus (%) = no. of eggs with pronucleus/total; no. of eggs embryo (%) = no. of embryos/no. of eggs with pronucleus.

**Figure 2:**
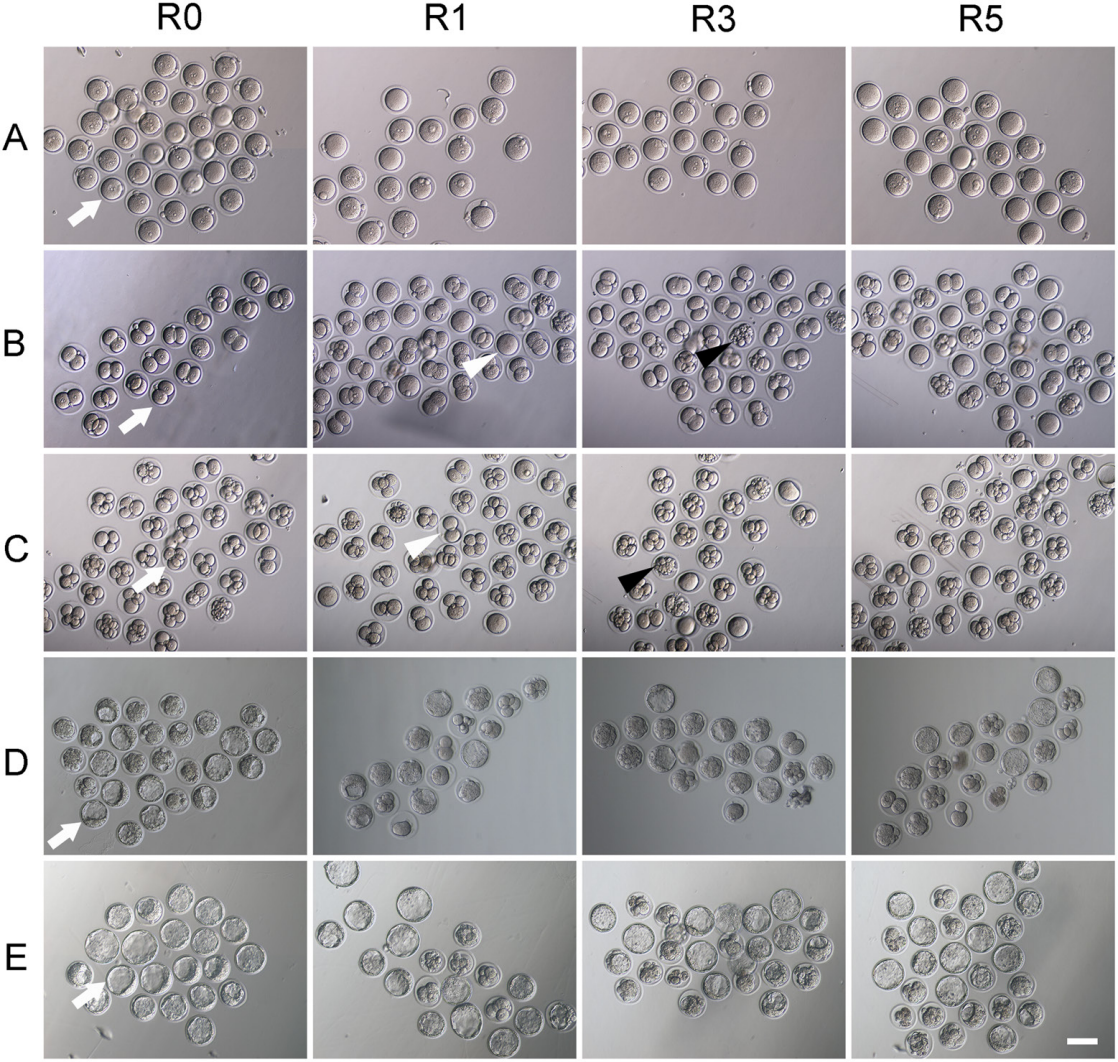
Representative images of *in vitro*-cultured embryos. Embryonic morphology images were acquired at 8, 24, 48, 84 and 108 h of culture. (A) Prokaryotic embryos at the PN4 phase; (B) two-cell stage embryos; (C) four-cell stage embryos; (D and E) blastocysts at 84 h and 108 h. Arrow indicates normal embryos at different stages, black arrowhead indicates dead embryos and white arrowhead refers to the blocked embryos. Scale bars = 100 μm.

The rate of pronucleus of R0 group was 91.3 ± 4.14%, while pronucleus rates were 50.4 ± 5.19%, 66.4 ± 6.15% and 42.7 ± 9.45% in R1, R3 and R5 groups, respectively. The cleavage rate was also significantly decreased with the increase of superovulation times (P < 0.05, Table 2). The incidence of two-cell embryos (Fig. 2B) in R5 group (85.4 ± 7.56%) was obviously lower than that in R0 group (97.9 ± 2.52%, *P* < 0.01), but it was similar between R1 group (91.9 ± 2.99%) and R3 group (91.7 ± 5.12%, *P* = 0.943). With the increase of superovulation times, the incidence of four-cell embryos had a significantly decreasing trend (*P* < 0.01). A similar trend was observed at the blastocyst stage (*P* < 0.01). The four-cell embryo rate of R0, R1, R3 and R5 groups were 94.6 ± 3.32%, 80.1 ± 5.37%, 77.0 ± 5.13% and 59.1 ± 9.31%, respectively. The frequency of blastocyst in R0 group was 82.9 ± 8.19%, which was significantly higher than that in R1, R3 or R5 group (52.0 ± 8.16%, 44.8 ± 10.46% and 37.7 ± 10.69%, respectively). Meanwhile, embryos derived from superovulation treatment had a higher incidence of death and developmental block during *in vitro* culture (Fig. 2 and Table 2). We also found that the cell number of blastocyst was significantly affected by superovulation, and ICM cell number was significantly decreased with the increase of superovulation times (*P* = 0.035153, Fig. 1D). To further understand the effects of superovulation on embryonic development, we investigated the expression of *Oct4*, *Sox2* and *Nanog*. The expression levels of these genes were similar among R1, R3 and R5 groups, although it was lower compared to R0 group (Fig. 1E). However, there was a decreased trend of these genes expression with the increase of superovulation times (Fig. 1E). The expression of genes related to apoptosis, such as *Bax*, *Bcl2* and *Casp 3* was not affected by superovulation in blastocysts (Fig. 1F). These results suggest that repeated superovulation may reduce embryonic development potential and embryonic quality in a superovulation time-dependent manner.

### Repeated superovulation altered histone acetylation level in the early embryos

**Figure 3:**
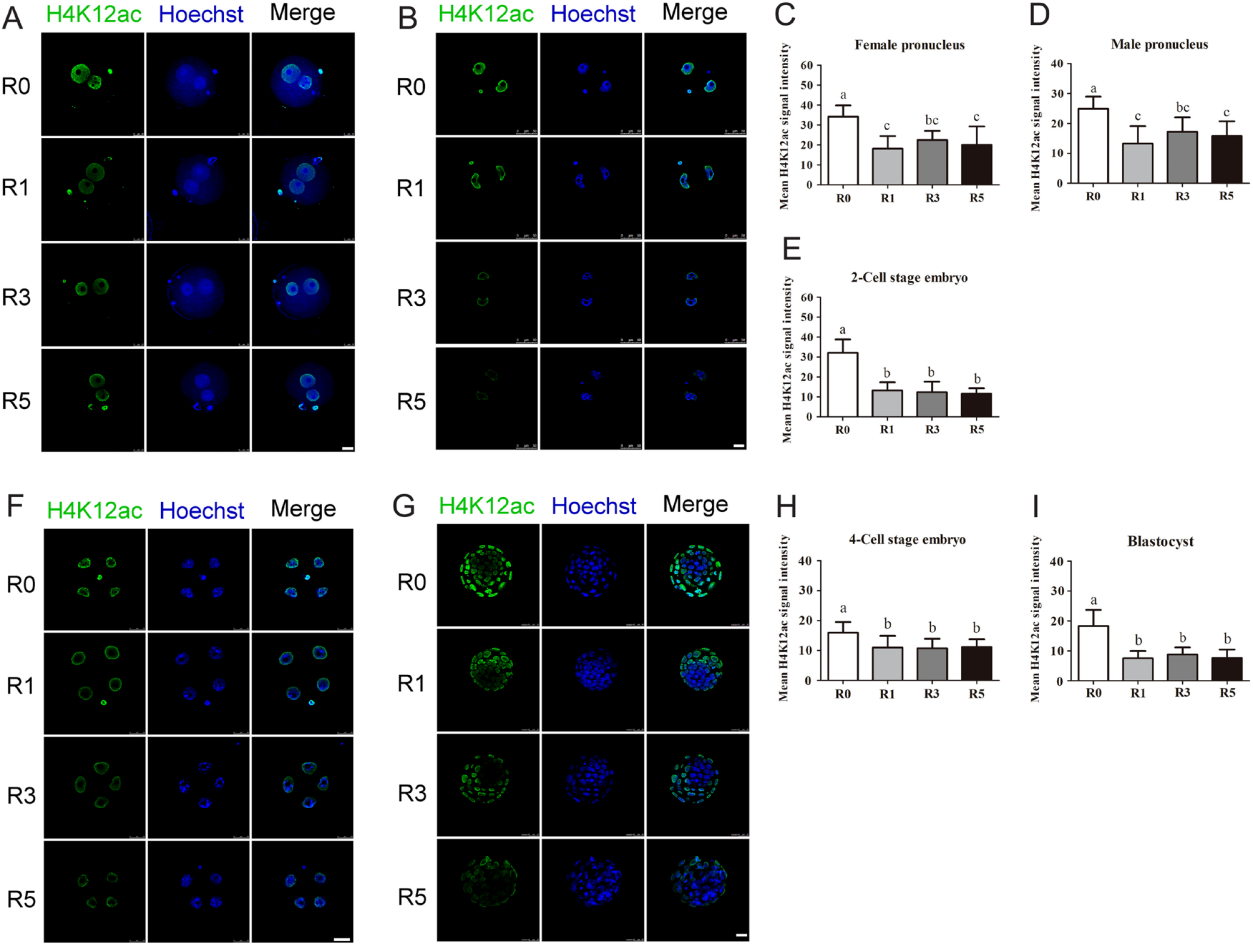
Acetylation levels of H4K12 in the early embryos. Fluorescence intensity of H4K12ac in early embryos was examined using confocal microscopy. (A) Pronuclear embryos at the PN4 phase, *n* = 42 (R0), *n* = 45 (R1), *n* = 44 (R3), *n* = 40 (R5); (B and C) average fluorescence intensity of pronucleus; (D) two-cell stage embryos, *n* = 52 (R0), *n* = 50 (R1), *n* = 57 (R3), *n* = 49 (R5); (E) average fluorescence intensity of two-cell stage embryos; (F) four-cell stage embryos, *n* = 39 (R0), *n* = 43 (R1), *n* = 45 (R3), *n* = 41 (R5); (H) blastocysts, *n* = 49 (R0), *n* = 47 (R1), *n* = 43 (R3), *n* = 45; (G and I) average fluorescence intensity of 4-cell stage embryos and blastocysts. Scale bars (prokaryotic embryos, four-cell stage embryos, blastocysts) = 25 μm. Scale bars (two-cell stage embryos) = 50 μm. Data present as mean ± s.d. Different letters indicate *P* < 0.05 between groups.

Proper histone modification is crucial for early embryo development, so we used immunofluorescence to examine the histone acetylation levels of H4K12ac and H4K16ac. The fluorescence intensity of H4K12ac was significantly stronger in R0 group than that in R1, R3 and R5 groups at the pronuclear stage (Fig. 3A). Similar results were observed at two-cell, four-cell and blastocyst stages (Fig. 3D, F and G). We further quantified the relative fluorescence intensity of H4K12ac and found that the relative fluorescence intensity of H4K12ac was lower in embryos of R1, R3 and R5 group compared to that of R0 group (*P* < 0.05), but there was no significant difference among R1, R3 and R5 groups (Fig. 3B, C, E, H and I).

**Figure 4:**
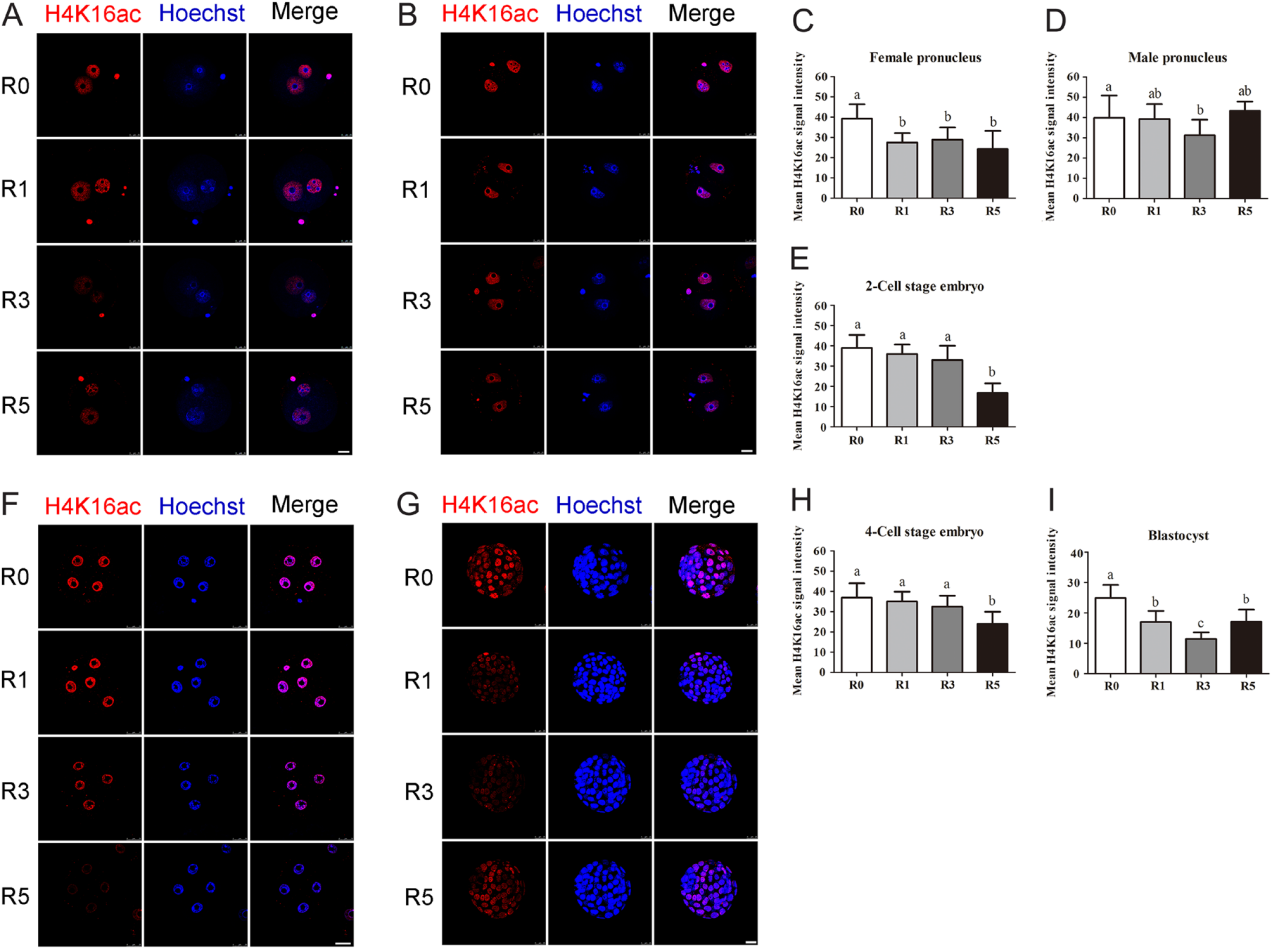
Acetylation levels of H4K16 in the early embryos. Fluorescence intensity of H4K16ac in early embryos was tested using confocal microscopy. (A) Pronuclear embryos at the PN4 phase, *n* = 44 (R0), *n* = 43 (R1), *n* = 42 (R3), *n* = 42 (R5); (B and C) average fluorescence intensity of pronucleus. (D) two-cell stage embryos, *n* = 50 (R0), *n* = 55 (R1), *n* = 52 (R3), *n* = 53 (R5); (E) average fluorescence intensity of two-cell stage embryos; (F) four-cell stage embryos, *n* = 37 (R0), *n* = 44 (R1), *n* = 50 (R3), *n* = 47 (R5); (H) blastocyst embryos, *n* = 50 (R0), *n* = 46 (R1), *n* = 47 (R3), *n* = 50; (G and I) average fluorescence intensity of four-cell stage embryos and blastocysts. Scale bars = 25 μm. Data present as mean ± s.d. Different letters indicate *P* < 0.05 between groups.

We found that H4K16ac was not affected by multiple superovulations in paternal pronucleus except for R3 group having a weaker fluorescence and relative low fluorescence intensity (*P* < 0.05; Fig. 4A and C). But the H4K16ac level was clearly decreased in maternal pronucleus (*P* < 0.05; Fig. 4C). At the two-cell and four-cell stages, the fluorescence level of H4K16ac in R5 group was obviously weaker than that in R0, R1 or R3 group (Fig. 4D and F). The relative fluorescence intensity of H4K16ac was also significantly lower in R5 group compared to that in R0, R1 or R3 group (*P* < 0.01; Fig. 4E and H). Blastocysts from superovulated mice had a lower H4K16ac level compared to that in R0 group (P < 0.01; Fig. 4G and I). These results indicate that superovulation obviously alters histone acetylation of H4K12 and H4K16 in preimplantation embryos and the effect of superovulation on histone acetylation of H4K16 shows a superovulation time-dependent manner.

### Repeated superovulation altered di-methylation of H3K9 and tri-methylation of H3K27 in the early embryos

**Figure 5:**
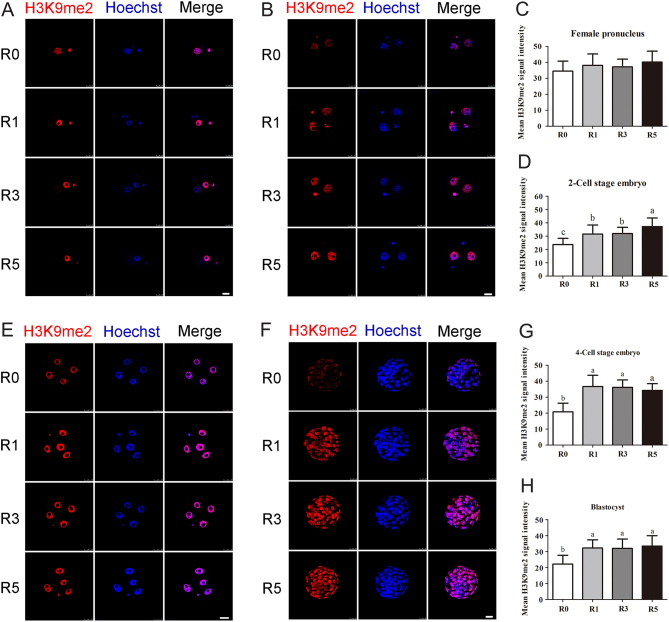
Di-methylation of H3K9 level in the early embryos. Fluorescence intensity of H3K9me2 in the early embryos was tested using confocal microscopy. (A) Pronuclear embryos at the PN4 phase, *n* = 47 (R0), *n* = 47 (R1), *n* = 49 (R3), *n* = 50 (R5); (B) average fluorescence intensity of pronucleus; (C) two-cell stage embryos, *n* = 51 (R0), *n* = 52 (R1), *n* = 52 (R3), *n* = 54 (R5); (D) average fluorescence intensity of two-cell stage embryos; (E) four-cell stage embryos, *n* = 39 (R0), *n* = 41 (R1), *n* = 44 (R3), *n* = 43 (R5); (F) average fluorescence intensity of four-cell stage embryos; (G) blastocysts, *n* = 47 (R0), *n* = 47 (R1), *n* = 47 (R3), *n* = 43; (H) average fluorescence intensity of blastocysts. Scale bars = 25 μm. Data present as mean ± s.d. Different letters indicate *P* < 0.05 between groups.

We further examined the effects of repeated superovulation on histone methylations. At the pronuclear phase, there was no difference of di-methylation of H3K9 (H3K9me2) level in maternal pronucleus among R0, R1, R3 and R5 groups (Fig. 5A and C). At the two-cell embryo stage, the H3K9me2 level was significantly increased with the increase of superovulation times (*P* < 0.05; Fig. 5B and D). Although H3K9me2 level was higher in 4-cell embryos and blastocysts, there was no difference among R1, R3 and R5 groups (*P* > 0.05; Fig. 5E, F, G and H).

**Figure 6:**
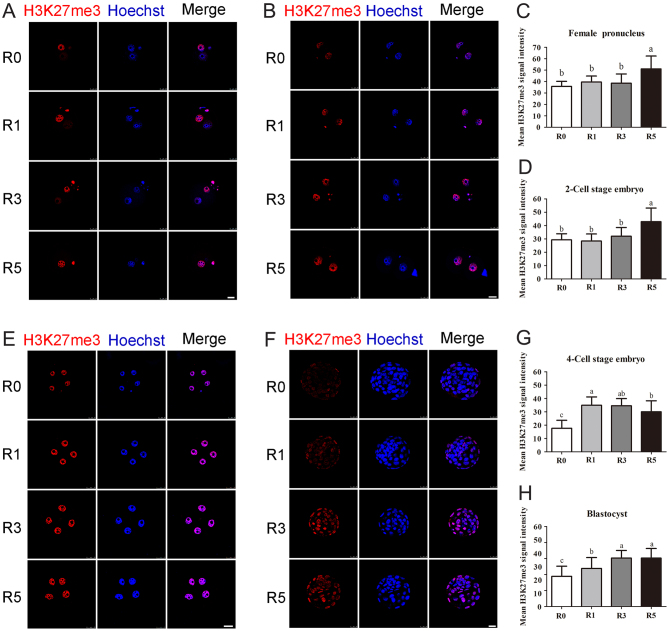
Tri-methylation of H3K27 level in the early embryos. Fluorescence intensity of H3K27me3 in the early embryos was examined using confocal microscopy. (A) Pronuclear embryos at the PN4 phase, *n* = 50 (R0), *n* = 50 (R1), *n* = 49 (R3), *n* = 50 (R5); (B) average fluorescence intensity of female pronucleus; (C) two-cell stage embryos, *n* = 53 (R0), *n* = 54 (R1), *n* = 54 (R3), *n* = 54 (R5); (D) average fluorescence intensity of two-cell stage embryos; (E) four-cell stage embryos, *n* = 44 (R0), *n* = 44 (R1), *n* = 44 (R3), *n* = 43 (R5); (F) average fluorescence intensity of four-cell stage embryos; (G) blastocysts, *n* = 46 (R0), *n* = 47 (R1), *n* = 46 (R3), *n* = 43; (H) average fluorescence intensity of blastocysts. Scale bars = 25 μm. Data present as mean ± s.d. Different letters indicate *P* < 0.05 between groups.

There was a significant increase for tri-methylation of H3K27 (H3K27me3) in R5 group at the pronucleus and two-cell embryo stages (*P* < 0.05; Fig. 6A, B, C and D). When embryos developed to the four-cell and blastocyst stages, the tri-methylation level of H3K27 was still higher in embryos experienced superovulation (*P* < 0.05; Fig. 6E, F, G and H), and the changes of the H3K27me3 level was at a superovulation time-dependent manner. It can be concluded from these results that superovulation has deleterious effects on H3K9me2 and H3K27me3 in early embryonic development. Furthermore, the effect of superovulation on H3K27me3 is in a superovulation-time-dependent manner.

### Multi-superovulation altered mRNA expressions of relative genes in the early embryos

We further examined mRNA expressions of genes encoding histone deacetylases HDAC1/2, acetylases GCN5 and HAT1, histone methyltransferases G9a and histone demethylases KDM6a/b, which regulate histone acetylation and methylation level at different stages of embryonic development.

**Figure 7:**
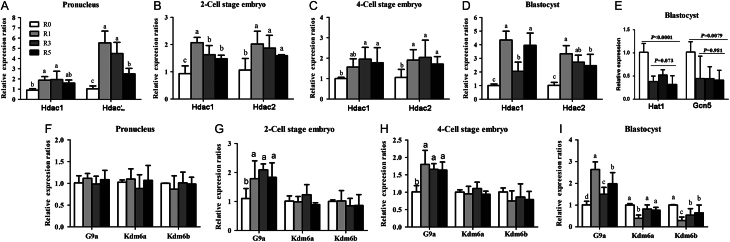
Expressions of *Hdac1*, *Ddac2*, *G9a*, *Kdm6a* and *Kdm6b* in the early embryos. The expressions of *Hdac1*, *Ddac2*, *G9a*, *Kdm6a* and *Kdm6b* in the early embryos at different stages were examined using qRT-PCR. (A) Expressions of *Hdac1* and *Hdac2* in the PN4 phase embryos (150 embryos, *n* = 3); (B) expressions of *Hdac1* and *Hdac2* in two-cell stage embryos (130 embryos, *n* = 3); (C) expressions of *Hdac1* and *Hdac2* in four-cell stage embryos (130 embryos, *n* = 3); (D) expressions of *Hdac1* and *Hdac2* in blastocysts (50 embryos, *n* = 3); (E) expressions of acetylases, such as Hat1 and Gcn5; (F) expressions of *G9a*, *Kdm6a* and *Kdm6b* in the PN4 phase embryos (150 embryos, *n* = 3); (G) expressions of *G9a*, *Kdm6a* and *Kdm6b* in two-cell stage embryos (130 embryos, *n* = 3); (H) expressions of *G9a*, *Kdm6a* and *Kdm6b* in four-cell stage embryos (130 embryos, *n* = 3); (I) expressions of *G9a*, *Kdm6a* and* Kdm6b* in blastocysts (50 embryos, *n* = 3). Data present as mean ± s.d. Different letters indicate *P* < 0.05 between groups.

At the pronuclear stage, the mRNA expression of both histone deacetylases *Hdac1* and *Hdac2* was higher in R1, R3 and R5 groups than in R0 group (*P* < 0.05; Fig. 7A). But the increased fold in R5 group was lower than that in R1 and R3 groups (Fig. 7A). At the two-cell embryo stage, the expression of *Hdac1* and* Hdac2* was also higher in R1, R3 and R5 groups than in R0 group, and the increased fold had a decreasing trend with increasing times of superovulation (*P* < 0.05; Fig. 7B). The higher expression level of *Hdac1* and *Hdac2* was maintained to the four-cell embryo (*P* < 0.05) and blastocyst (P < 0.05) stages. There was also a decreased trend of the mRNA expression level of *Hdac1* and *Hdac2* with increased superovulation times in four-cell embryos and blastocysts though the difference was not significant (*P* > 0.05; Fig. 7C and D). Meanwhile, we found that the expression of *Gcn5* and* Hat1* in blastocysts was similar among R1, R3 and R5 groups, but it was lower when compared to that in R0 group (Fig. 7E). These indicate that deacetylases may play a key role in the decrease of histone acetylation with the increase of superovulation times.

There was no significant change in the expression of *G9a*, *Kdm6a* and *Kdm6b* among the R1, R3 and R5 groups at the pronuclear stage (*P* > 0.05; Fig. 7F). At the two-cell and four-cell embryo stages, the expression of *G9a* was significantly increased in R1, R3 and R5 groups compared to that of R0 group (*P* < 0.05; Fig. 7G and H). The expression of *Kdm6a* and *Kdm6b* was not affected by superovulation (Fig. 7G and H) at the two-cell and the four-cell embryo stages. In blastocysts, superovulation increased the expression of *G9a* in R1, R3 and R5 groups, but with the increase of superovulation times, there was a significantly decreased trend of *G9a* expression, especially for R3 group (*P* < 0.05; Fig. 7I). The expression of *Kdm6a* and *Kdm6b* was reduced by superovulation in blastocysts, especially *Kdm6b* in R1, R3 and R5 groups (*P* < 0.05; Fig. 7I). These results indicate that multi-superovulation alters histone modifications by influencing the expression of histone deacetylases, methyltransferase and demethylases in mouse early embryos.

## Discussion

Superovulation is one of the most important technologies in ART and is widely used in human and animals to get more available oocytes. In the clinic, many women experience more than one exogenous hormone stimulation cycle to obtain a healthy baby. Similarly, repeated superovulation is also used in animals to increase the utilization rate of good females. But studies have shown that ovarian stimulation has an adverse effect on granulosa cell apoptosis (Tarin *et al.* 2001, Combelles & Albertini 2003) and mitochondrial copy number in cumulus cells (Xie *et al.* 2016). Superovulation can also lead to changes in cytoplasmic distribution of organelles in oocytes, spindle abnormalities, abnormal expression of octamer-binding transcription factor 4 (Oct4) and a decrease of oocyte development potential (Kalthur *et al.* 2016). Van Blerkom and Davis confirmed that repeated ovarian stimulation in mice significantly increases the frequency of spindle defects and causes chromosome errors (Wilding *et al.* 2001). Superovulation also alters nuclear maturation, cAMP in oocytes, ultrastructure of oocytes and the expression of *Epab* and *Pabpc1* (Hyttel *et al.* 1989, Wang *et al.* 2006, Dadarwal *et al.* 2015, Ozturk *et al.* 2016). Furthermore, a recently published paper demonstrated that repeated superovulation decreases 5-methylcytosine level in mouse oocytes (Xiao *et al.* 2019). The compromised oocyte quality may be an important reason for the decrease in the percentage of oocytes with the 1st PB and the rate of pronucleus at a superovulation time-dependent manner. For example, a recent study found repeated superovulation disturbs spindle organization and chromosome alignment during oocyte maturation (Xiao *et al.* 2019). Previous studies showed that superovulation increased the percentage of immature oocytes and decreased the fertilization rate, pronuclear rate and embryonic developmental potential (Ishibashi & Aoki 1977, Evans & Armstrong 1984, Sartori *et al.* 2010, Kon *et al.* 2014, Taiyeb *et al.* 2017). To avoid the effects of males on embryonic development, we used the same WT males in our experiments in each group. Our data also suggest that multiple superovulations decrease early embryonic development potentials, such as a lower blastocyst rate and a higher incidence of arrested embryos. In the human being, ART increases the risk of miscarriage, preterm delivery and low birth weight (De Geyter *et al.* 2006), which suggests that embryonic development potential may be compromised by ART manipulation. These results demonstrate that superovulation decreases the embryonic developmental potential.

To activate the zygotic genome, chromatin structure remodeling is required after fertilization. Epigenetic modifications play a pivotal role in regulating chromatin structure (Eckersley-Maslin *et al.* 2018). It is demonstrated that zygotic genome activation (ZGA) is regulated by histone modifications, miRNAs, DNA methylation, and so on (Zenk *et al.* 2017, Eckersley-Maslin *et al.* 2018). H4K12ac is an important histone modification in sperm and it is inherited by paternal pronucleus after fertilization (van der Heijden *et al.* 2006). For the maternal pronucleus, there is an increase of H4K12ac until the fusion of maternal and paternal pronuclei (Vieweg *et al.* 2015). H4K12ac is enriched at CTCF-binding sites and transcription start sites of genes involved in the developmental processes (Arpanahi *et al.* 2009) and gene activation in early embryos (Paradowska *et al.* 2012). For example, genes activated by H4K12ac at the four-cell embryo stage are mainly associated with gene expression, histone fold and DNA-dependent transcription. Genes activated by H4K12ac at the eight-cell embryo and blastocyst stage are involved in developmental processes (Paradowska *et al.* 2012). H4K16ac is another crucial acetylation at lysine 16 of histone 4 which can disrupt high-order chromatin structure and activate gene transcription *in vivo* and *in vitro* (Wu *et al.* 2011). H4K16ac is also very important for embryogenesis in humans and mice (Gupta *et al.* 2008, Lin *et al.* 2013). Histone deacetylases of HDAC1 and HDAC2 are important enzymes regulating histone acetylation and early embryo development (Ma & Schultz 2016). When fertilized embryos are treated with an inhibitor of HDAC, the blastocyst rate is significantly reduced (Ma *et al.* 2001). In the present study, we tested the mRNA expressions of *Hdac1* and *Hdac2* in embryos at different stages and found that the expression of* Hdac1* and *Hdac2* was higher in the early embryos from superovulation groups. This indicates that the abnormal expression of *Hdac1* and *Hdac2* may play a key role in the reduced H4K12ac and H4K16ac levels in early embryos.

Histone methylation is another important histone modification which plays an important role in the early embryos. H3K9me2 widely exists in the genome and undergoes great changes in cell differentiation (Lienert *et al.* 2011, Chen *et al.* 2012). In preimplantation mouse embryos, H3K9me2 has an important contribution to silencing retrotransposon to protect the genomes (Hatanaka *et al.* 2015). PGC7 suppresses the conversion of 5mC to 5hmC in early embryos via binding to histone H3K9me2, too (Nakamura *et al.* 2012). We found that repeated superovulation increased H3K9me2 in early embryos, which may be caused by the increase of G9a (Tachibana *et al.* 2002). H3K27me3 locates at the promoter regions of genes involved in developmental processes and represses genes’ expression in the early embryos (Shpargel *et al.* 2012). In early embryo development, there is a dynamic change of H3K27me3 at different stages. In blastocyst, H3K27me3 is lower than in other stage embryos (Liu *et al.* 2016). But the signal of H3K27me3 in early embryos derived from superovulation was significantly higher compared to that of non-stimulated mouse embryos. The increase of H3K27me3 was in a superovulation time-dependent manner. The dynamic change of H3K27me3 in the early embryos is mainly regulated by histone demethylases KDM6 and JmjC (Jumonji-C) (Shpargel *et al.* 2012). Therefore, the decreased expression of Kdm6a and Kdm6b may be essential for the higher signal of H3K27me3 in the early embryos.

Our data suggest that the alteration of histone modifications in early embryos may affect chromatin structure remodeling, which regulates ZGA. After fertilization, ZGA is the most important event as it initiates embryonic development. Embryos would be blocked at the two-cell stage in mice if the zygotic genome is not activated after fertilization. If ZGA is compromised, it would decrease embryonic development potentials, such as a lower blastocyst rate and a higher incidence of blocked embryos (Jachowicz *et al.* 2017). Therefore, it is plausible that multiple superovulations may affect embryonic quality and development through influencing chromatin structure and ZGA.

In summary, we found that repeated superovulations altered histone modifications in early embryos via increasing the expressions of *Hdac1*, *Hdac2* and *G9a* and decreasing the expressions of *Kdm6a* and *Kdm6b*. The alteration of histone modifications may play a pivotal role in compromised embryonic development. However, it is not clear how multiple superovulations regulate gene expressions and histone modifications. Furthermore, what pathways mediate the alteration of histone modifications in this model remains ambiguous. It is demonstrated that the ovarian structure and mitochondrial function in cumulus cells are affected by multiple superovulations. Therefore, the dysfunction of mitochondria in cumulus cells might play an important role in mediating the effects of multiple superovulations on epigenetic marks. More studies are needed to investigate the mechanism underlying the effect of repeated superovulations on embryonic development and offspring health.

## Declaration of interest

The authors declare that there is no conflict of interest that could be perceived as prejudicing the impartiality of the research reported.

## Funding

This work was supported by the funds of the National Natural Science Foundation of China (81401198, 31872312) and High-level Personnel Scientific Research Fund of Qingdao Agricultural University (1116008, 6631113337).

## Author contribution statement

Tang S B designed the study, analyzed data and wrote the manuscript; Yang L L organized the charts; Zhang T T interpreted the data; Yin S, Luo S M and Shen W participated in the conception of study design and revised the manuscript; Ge Z J and Sun Q Y conceived the study design, participated in data analysis and interpretation.
